# The protective role of ginsenoside Rg3 in heart diseases and mental disorders

**DOI:** 10.3389/fphar.2024.1327033

**Published:** 2024-02-26

**Authors:** Lili Shi, Jinlan Luo, Xiupan Wei, Xizhen Xu, Ling Tu

**Affiliations:** ^1^ Department of Geriatric Medicine, Tongji Hospital, Tongji Medical College, Huazhong University of Science and Technology, Wuhan, China; ^2^ Hubei Key Laboratory of Genetics and Molecular Mechanisms of Cardiological Disorders, Wuhan, China; ^3^ Department of Rehabilitation Medicine, Zhongda Hospital, Southeast University, Nanjing, China; ^4^ Division of Cardiology and Department of Internal Medicine, Tongji Hospital, Tongji Medical College, Huazhong University of Science and Technology, Wuhan, China

**Keywords:** ginsenoside Rg3, heart diseases, mental disorders, heart failure, depression

## Abstract

Ginsenoside Rg3, a compound derived from *Panax ginseng* C. A. Mey., is increasingly recognized for its wide range of pharmacological effects. Under the worldwide healthcare challenges posed by heart diseases, Rg3 stands out as a key subject in modern research on Chinese herbal medicine, offering a novel approach to therapy. Mental illnesses are significant contributors to global disease mortality, and there is a well-established correlation between cardiac and psychiatric conditions. This connection is primarily due to dysfunctions in the sympathetic-adrenomedullary system (SAM), the hypothalamic-pituitary-adrenal axis, inflammation, oxidative stress, and brain-derived neurotrophic factor impairment. This review provides an in-depth analysis of Rg3’s therapeutic benefits and its pharmacological actions in treating cardiac and mental health disorders respectively. Highlighting its potential for the management of these conditions, Rg3 emerges as a promising, multifunctional therapeutic agent.

## 1 Introduction

Despite extensive research over the years, both heart diseases and mental disorders continue to exert significant pressure on global healthcare systems. Heart diseases maintain their status as the leading cause of death globally (Liu and Miao, 2022; Lupisella et al., 2022). The pharmacological landscape for heart diseases is diverse, encompassing a range of drugs. However, these treatments largely fail to restore cardiac function fundamentally and are frequently associated with side effects.

At the same time, mental illness significantly contributes to the global disease burden. A recent meta-analysis reveals that 14.3% of global deaths annually, equivalent to roughly eight million fatalities, are linked to mental disorders (Walker et al., 2015). Individuals with severe mental illnesses, such as schizophrenia, bipolar disorder, and major depressive disorder (MDD), face a mortality rate of two to three times higher than the average population. The elevated rate corresponds to a reduced life expectancy of 10–25 years (Correll et al., 2015). However, issues associated with treatment discontinuation and ineffectiveness are prevalent.

Up to now, numerous studies have established a strong link between cardiac diseases and psychiatric conditions. It is frequently observed that individuals with cardiac ailments often experience psychiatric disturbances (Piepenburg et al., 2019). Inversely, those with mental disorders appear to have a higher risk of developing heart diseases (Hagi et al., 2021). Several biological mechanisms are suggested to clarify the association between mental disorders and cardiac events. Mental disorders are linked to dysfunctions in the sympathetic-adrenomedullary system (SAM), and the hypothalamic-pituitary-adrenal (HPA) axis, as well as to inflammation, oxidative stress, and impairments in the brain-derived neurotrophic factor (BDNF) system. All these physiological processes play significant roles in the onset and progression of cardiac diseases.

Recently, the field of Chinese herbal medicine has captured the interest of the scientific community, owing to its extensive range of pharmacological properties and a lower incidence of adverse side effects. Particularly, the effects of ginsenoside Rg3 (Rg3) in cardiac and mental diseases have gained more and more attention.

Consequently, in this review, we provide a detailed investigation of the therapeutic effects and pharmacological action of Rg3 in addressing heart and mental disorders (Table 1). Based on the association between heart and mental disorders, we aim to provide prospects on the potential effects and mechanisms of Rg3 in the comorbid conditions.

**TABLE 1:** Summary of effects of Rg3 on heart and mental conditions.

Disease	Model	Type	Treatment (dose, duration)		Described effects and mechanisms	References (PMID)
			Animal	Cell		
HF	TAC-mice	20(R)-Rg3	20 mg/kg	10 μM	Improving cardiac function, inhibiting cardiomyocyte hypertrophy and ER stress via enhancing SUMOylation of SERCA2a	34,428,586
	ISO-HL-1 cell		4 weeks	24 h		
	TAC-mice	—	10 or 20 mg/kg	10 μM	Modulating glucose metabolism and insulin resistance through activation of the AMPK pathway	35,509,823
	Insulin-H9C2		4 weeks	24 h		
	TAC-mice	—	10 or 20 mg/kg	—	Regulating pyruvate metabolism and sustaining glucose homeostasis in cardiac tissue through maintaining the PDHc activity	36,572,672
			4 weeks			
	CAL-mice	20(S)-Rg3	7.5, 15 or 30 mg/kg	1, 5 or 25 µM	Inhibiting myocardial fibrosis via the ACY1/TGFβ1/Smad3 signaling pathway	34916608
	Ang II-CFs		2 weeks	24 h		
	LAD-rat	—	10, 20 or 30 mg/kg	10 µm	Activating mitophagy via the ULK1/FUNDC1 pathway	37,659,296
	H9C2		4 weeks	20 h		
MI	CAL-rat	—	30 mg/kg/day	—	Inhibiting the inflammatory response via the NF-κB pathway	33193843
			1 week			
	ISO-mice	—	5 mg/kg	—	Upregulating autophagy process through the AMPK signaling pathway	32420095
			2 days			
	LAD-mice	20(S)-Rg3	20 or 40 mg/kg	5, 10 or 20 µM	Alleviating myocardial fibrosis through the TGFBR1 signaling pathway	38107395
	TGFβ1-CFs		4 weeks			
MIRI	Rat	—	5 or 20 mg/kg	—	Antiapoptosis and anti-inflammation	28105061
			7 days			
	Rat	—	0.5 mg	10 nM	Antifibrosis, inhibiting oxidative stress and inflammation via SIRT1/PGC1-α/Nrf and IκBα/NF-κB signal pathway	31,783,047
	H9C2			24 h		
	Rat	—	60 mg/kg	10 mM	Inhibiting apoptosis and oxidative stress via Akt/eNOS signaling and the Bcl2/Bax pathway	25,672,441
	NRCs			24 h		
Cardiotoxicity	ADM-rats	—	10, 20 or 40 mg/kg	1, 10 or 100 µM	Inhibiting oxidative stress via activation of the Nrf2-ARE pathway through the activation of Akt	26,321,736
	CMEC		2 weeks	24 h		
	Mice	—	10, 40 or 80 mg/kg	60 μM	Inhabiting apoptosis and oxidative stress through the miR-128–3p/MDM4 axis	37,990,515
	H9C2		4 weeks	25 h		
DCM	Mice	—	25, 50 or 100 mg/kg	5, 10 or 20 μM	Modulating glucose and lipid metabolism by directly binding to PPAR-γ and activation of the adiponectin pathway	38,069,059
	H9C2		12 weeks	48 h		
	3T3-L1					
MDD	LPS-mice	—	20 or 40 mg/kg	—	Inhibition of neuroinflammatory disturbance and the regulation of TRP-KYN metabolism	28,762,741
			3 days			
	Mice	—	10 or 20 mg/kg	—	Promotion of the BDNF signaling pathway	28,013,484
			2 weeks			
	Mice	—	50, 100 or 150 mg/kg	1, 5 or 10 μM	Recovering proliferation and inhibiting apoptosis via CREB and BDNF signaling pathway	28,461,003
	NMDA-HT22		4 weeks			
Anxiety	*Xenopus* oocytes	20(S)-Rg3	—	100 μM	Regulating GABA_A_ receptor channel activity	23,499,684
PTSD	Rat	—	25 or 50 mg/kg	—	Regulating the HPA axis and activating the BDNF-TrkB pathway	35,982,366
			2 weeks			
ADHD	Mice	YY162(including 20(R)-Rg3 and 20(S)-Rg3)	200 mg/kg	100 μg/mL	Inhibiting oxidative stress	24,394,491
	SH-SY5Y		2 weeks	50 h		

TAC, transverse aortic constriction; ISO, isoproterenol; ER, endoplasmic reticulum; SERCA2a, sarcoplasmic/endoplasmic reticulum Ca^2+^-ATPase; AMPK, AMP-activated protein kinase; PDHc, pyruvate dehydrogenase complex; CAL, coronary artery ligation; AngII, angiotensin II; ACY1, aminoacylase-1; TGFβ1, transforming growth factor-β 1; LAD, left anterior descending coronary artery ligation; ULK1, Unc51-like-kinase 1; FUNDC1, FUN14 domain-containing protein 1; NFκB, nuclear factor κB; TGFBR1, transforming growth factor beta receptor 1; PGC1-α, peroxisome proliferators-activated receptor γ coactivator l alpha; IκBα, inhibitor of kappa B alpha; Nrf2, nuclear factor erythroid 2-related Factor 2; Akt, protein kinase B; eNOS, endothelial nitric oxide synthase; Bcl-2, B cell lymphoma-2; Bax, Bcl2-associated X protein; ARE, antioxidant response element; MDM4, double minute 4 protein; PPAR-γ, peroxisome proliferator-activated receptor γ; TRP, tryptophan; KYN, kynurenine; BDNF, brain-derived neurotrophic factor; CREB, cyclic adenosine monophosphate response element binding protein; HPA, hypothalamic-pituitary-adrenal; TrkB, tropomyosin-related kinase B; LPS, lipopolysaccharide; HT22, murine hippocampal neuronal; NMDA, n-methyl-d-aspartate; HF, heart failure; MI, myocardial infarction; MIRI, myocardial ischemia-reperfusion injury; MDD, major depressive disorder; DCM, diabetic cardiomyopathy; PTSD, post-traumatic stress disorder; ADHD, attention deficit hyperactivity disorder.

## 2 Origin and structure of Rg3

**FIGURE 1:**
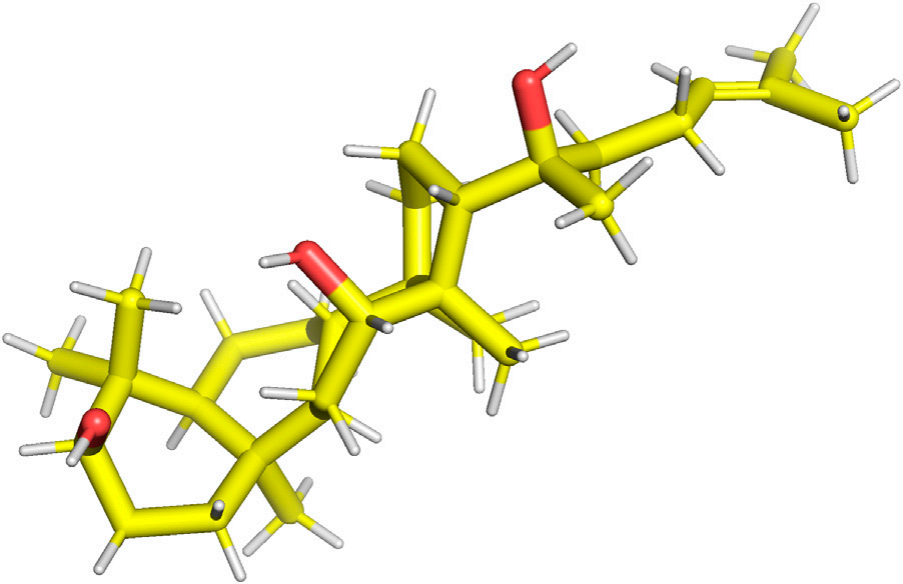
Structure of 20(R)-Rg3.

**FIGURE 2:**
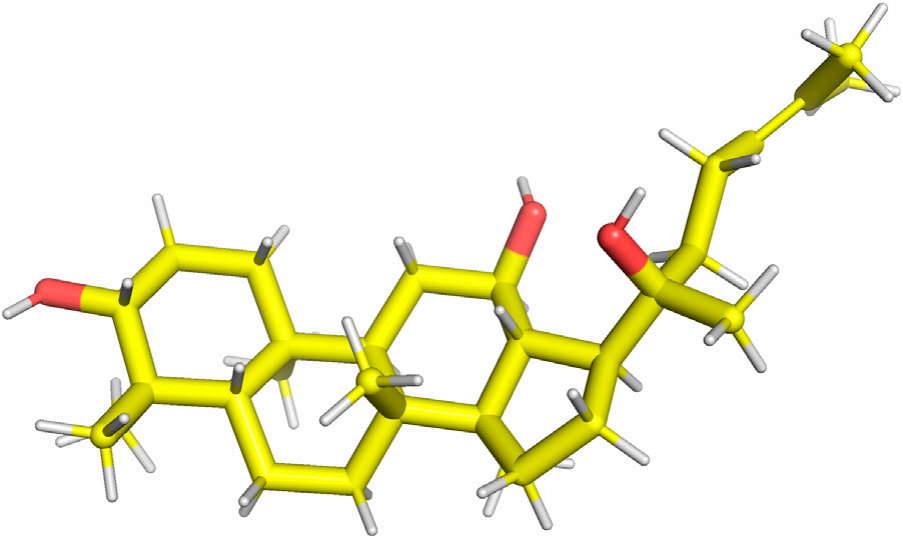
Structure of 20(S)-Rg3.

Rg3 is a constituent of ginsenosides extracted from *Panax ginseng* C. A. Mey. (Nakhjavani et al., 2020). Key ginsenoside constituents, specifically Rb1, Rb2, and Rd, possess the capacity for enzymatic transformation into Rg3 (Lee H. et al., 2020). Rg3 is categorized into two distinct stereoisomers based on its unique spatial configurations at the C20 position: 20(R)-Rg3, as illustrated in Figure 1, and 20(S)-Rg3, depicted in Figure 2. Research findings have underscored the pivotal role of Rg3 across diverse domains, encompassing its involvement in anti-aging mechanisms, anticancer properties, bone development, cellular differentiation, neuroprotection, and cardiac function (Lee et al., 2019; Zarneshan et al., 2022).

## 3 The relationships between heart diseases and mental disorders

### 3.1 Mental disorders induce the occurrence of heart diseases

Recent meta-analytic studies have shown that mental illness is a high risk for cardiac events (Zarneshan et al., 2022). In addition, a Mendelian randomization analysis revealed a significant genetic association between MDD and coronary artery disease (CAD). Specifically, the analysis indicated that for each one-unit increase in the natural logarithm of odds of MDD, the odds ratio for CAD was 1.16 (95% confidence interval: 1.05 to 1.29; *p* = 0.0047) (Tang et al., 2020). Recent observational research has indicated a correlation between genetic predispositions for schizophrenia and notable changes in cardiac structure. These alterations have been found to potentially aggravate cardiac health outcomes (Pillinger et al., 2023). Mental health disorders, such as MDD, anxiety, and stress-related conditions, may contribute to behaviors including smoking, inactivity, and drinking adversely affecting cardiac function.

### 3.2 Heart diseases promote the development of mental disorders

Clinically, a subset of patients with heart diseases commonly experience co-occurring mental disorders, specifically anxiety and depression. Multiple meta-analyses have demonstrated that individuals diagnosed with heart failure (HF) are at an elevated risk of experiencing depression (Hagi et al., 2021). Certain drugs used in managing heart diseases, including beta-blockers, have the potential to initiate or aggravate symptoms associated with anxiety and depression (Bornand et al., 2022; Carnovale et al., 2023). Additionally, managing heart conditions, including HF, entails a prolonged treatment course that can result in significant financial strain for patients and create a disparity between hospitalization needs and employment stability. This significantly heightens the risk of developing mental disorders in individuals with heart diseases.

### 3.3 The major mechanisms linking heart diseases and mental disorders

#### 3.3.1 Inflammation

Inflammatory processes are associated with the initiation and progression of mental disorders (Ben-Azu et al., 2022a; Ben-Azu et al., 2023). Mental disorders may enhance the expression of pro-inflammatory cytokines, such as interleukin-6 (IL-6) and tumor necrosis factor-alpha (TNF-ɑ), which are associated with endothelial dysfunction and metabolic alterations (Vaccarino et al., 2018; Misiak et al., 2022; Tsioufis et al., 2022; Chen et al., 2023). Inflammation, vascular dysfunction, and metabolic abnormalities frequently contribute to the pathogenesis of heart diseases. Furthermore, inflammatory processes within heart tissue can result in elevated blood levels of pro-inflammatory cytokines and other acute-phase reactants (Hartupee and Mann, 2017). Inflammatory factors may lead to the upregulation of indoleamine 2,3-dioxygenase (IDO), diverting tryptophan (TRP) into the kynurenine (KYN) pathway, potentially reducing serotonin (5-HT) synthesis and contributing to the onset of mental disorders, including depression. 5-HT functions as a neurotransmitter in the central nervous system, a blood factor, and a neurohormone that regulates the function of various peripheral organs (De Deurwaerdere and Di Giovanni, 2020). A deficiency in 5-HT increases susceptibility to social defeat stress and impairs responses to antidepressants (Sachs et al., 2015). Additionally, pro-inflammatory cytokines are associated with a marked decrease in both BDNF gene and protein expression (Zhang et al., 2014). BDNF is crucial for the plasticity of glutamatergic and gamma aminobutyric acid (GABA)ergic synapses and is intimately linked to severe mental illnesses (Colucci-D'Amato et al., 2020). Interestingly, animal studies have demonstrated that in post-myocardial infarction, BDNF expression is upregulated through neuronal signaling originating from the heart. This upregulation serves to shield the myocardium from ischemic damage, thereby exerting a protective effect against cardiac remodeling (Cannavo et al., 2023). Furthermore, studies have noted lower BDNF levels in patients with HF when compared to healthy controls (Xie et al., 2023; Wang et al., 2024). BDNF plays a crucial role in supporting the survival of endothelial cells during the development of the cardiovascular system (Kermani and Hempstead, 2019).

#### 3.3.2 HPA axis

The HPA axis serves as a critical component of the neuroendocrine system, orchestrating responses to both internal and external stressors. Various mental disorders have been shown to trigger the activation of the HPA axis (Sur and Lee, 2022b; Emudainohwo et al., 2023; Menke, 2024). This activation leads to an upsurge in cortisol synthesis within the adrenal cortex, along with enhanced production of adrenaline and noradrenaline in the adrenal medulla (Ugwu et al., 2022). Hypercortisolemia, commonly observed in mental disorders, can lead to escalated steroid production, elevated blood pressure, and an increase in visceral fat (García-Eguren et al., 2019; Favero et al., 2021; Teng et al., 2021). These changes significantly heighten the risk of heart diseases. Elevated cortisol levels following stress are directly linked to the hypertrophy of cardiomyocytes and cardiac remodeling (Opinion et al., 2023). Additionally, plasma cortisol concentrations have been identified as an independent risk factor for cardiac events and mortality (Crawford et al., 2019; Kim et al., 2022). Furthermore, the stimulation of the HPA axis results in increased aldosterone levels. Studies have shown that stress-related aldosterone activity has been linked to hypertension, myocardial necrosis, and fibrosis. Additionally, a rise in aldosterone has been associated with increased insulin resistance, oxidative stress, and pro-inflammatory responses (Bothou et al., 2020; Tsai et al., 2021; Campana et al., 2022). In chronic HF, elevated serum cortisol levels have been identified as an independent predictor of increased mortality risk (Güder et al., 2007). Moreover, increased cortisol levels may exacerbate the brain’s vulnerability to oxidative stress, potentially leading to detrimental effects on neurobehavioral health. Stress-induced cortisol secretion may lower brain 5-HT and BDNF function, potentially leading to the onset of depressive symptoms and anxiety (Bhagwagar et al., 2002; Motta et al., 2021).

#### 3.3.3 SAM

Negative psychological states can activate the SAM system, resulting in elevated catecholamine levels. Catecholamines, including epinephrine, norepinephrine, and dopamine, are tyrosine-derived hormones and neurotransmitters primarily synthesized in the adrenal medulla, sympathetic nerves, and brain. Elevated catecholamine levels can induce vasoconstriction, leading to cardiac injury, HF, myocardial ischemia, and necrosis (Szatko et al., 2023). Persistent catecholamine elevation may cause myocardial calcium overload in cytosolic and mitochondrial compartments, trigger oxidative stress, increase mitochondrial permeability, and cell death (Szatko et al., 2023). In patients with HF, there is an activation of the neuroendocrine systems, particularly the sympathetic nervous system and the renin-angiotensin-aldosterone system, leading to elevated levels of neurohormones such as catecholamines (Manolis et al., 2023). Dopamine, an important catecholamine, plays a crucial role in regulating various mental and physical functions, including anxiety, fear, attention deficit hyperactivity disorder (ADHD), and schizophrenia (Jayanti et al., 2023). Additionally, norepinephrine, another key catecholamine, acts as a neurotransmitter and is essential in mediating physiological and behavioral responses to stress (Schmidt et al., 2019).

## 4 Pharmacological action of Rg3 in heart diseases

### 4.1 HF

HF is a complicated condition that leads to aggressive hazards to human health. The common therapeutic regimens for HF patients predominantly involve diuretics, vasoactive drugs, and other pharmaceutical approaches (Truby and Rogers, 2020). Medications such as angiotensin-converting enzyme inhibitors (ACEIs), beta-blockers, mineralocorticoid receptor antagonists, and angiotensin receptor-neprilysin inhibitors can partially alleviate HF symptoms. However, these treatments are often insufficient in significantly reducing rehospitalization rates and mortality, even when patients adhere to established guidelines (Lai et al., 2022). Moreover, traditional medication can lead to adverse effects, including hypotension, hypokalemia, and renal function impairment (Hubers and Brown, 2016; Marciniak and Serebruany, 2019; Rossello et al., 2022). Consequently, there is a pressing need to explore new therapeutic agents to enhance survival rates and improve the quality of life for HF patients. Rg3 has emerged as a promising treatment option in HF, offering protective benefits such as promoting cardiomyocyte relaxation, enhancing mitochondrial structure and function, regulating metabolism, reducing cardiac fibrosis, and preventing cell apoptosis.

Cardiac sarcoplasmic/endoplasmic reticulum Ca^2+^-ATPase (SERCA2a), a crucial protein in the Ca^2+^ cycle of cardiomyocytes, is involved in Ca^2+^ reuptake into the cytoplasm and subsequent transport to the endoplasmic reticulum (ER), ultimately promoting cardiomyocytes relaxation (Liu et al., 2021; Chen et al., 2022). SUMO binds to certain lysine sites on SERCA2a, forming the SERCA2a/SUMO complex. Notably, HF patients and mice exhibited significantly reduced levels of SERCA2a SUMOylation (Mendler et al., 2016). However, Rg3 treatment could increase the SUMOylation of SERCA2a, further increase intracellular Ca^2+^ cycle protein levels, suppress ER stress and prevent reaction oxygen species (ROS) generation, thus improving cardiac function and inhibiting cardiomyocyte hypertrophy in transverse aortic constriction (TAC) induced HF mice (Liu et al., 2021).

Moreover, Rg3 could improve disordered mitochondrial ultrastructure, functions such as ATP production and spare respiratory capacity, and regulate glucose uptake, and myocardial insulin resistance (Ni et al., 2022a). Rg3 regulated glucose uptake and myocardial insulin resistance through the activation of insulin receptor substrate (IRS)-phosphoinositide 3 kinase (PI3K)-protein kinase B (Akt) signaling pathway (Ni et al., 2022a). Several pieces of experimental evidence suggested that the decoupling of glucose oxidation to glycolysis may be the cause of unaltered or reduced pyruvate oxidation in mitochondria in HF (Pound et al., 2009; Bertero and Maack, 2018; Fillmore et al., 2018). Pyruvate dehydrogenase complex (PDHc) plays a pivotal role in regulating mitochondrial pyruvate metabolism with dihydrolipoamide dehydrogenase (DLD), serving as a crucial part of PDHc (Staretz-Chacham et al., 2021; Kim et al., 2023). P300 and tat-interacting protein 60 (TIP60) are recognized as 2-hydroxyisobutyryltransferases that could regulate the activity of PDHc (Sabari et al., 2017; Huang et al., 2018). In HF, the 2-hydroxyisobutylation of DLD was significantly upregulated, resulting from the downregulation of PDHc activity. However, Rg3 can lower the 2-hydroxyisobutylation levels of DLD and maintain the PDHc activity by suppressing the acyltransferase activity of P300, further regulating pyruvate metabolism and sustaining glucose homeostasis in cardiac tissue, consequently, improving cardiac function (Ni et al., 2022b).

Another study demonstrated that in HF mice, Rg3 administration increased the expression of aminoacylase-1 (ACY1) and inhibited cardiac fibrosis, thereby, ameliorating heart function through the ACY1-mediated transforming growth factor-β1 (TGF-β1)/Smad3 pathway. In this study, metoprolol served as the positive control. Both Rg3 and metoprolol significantly enhanced cardiac function. Notably, the impact of Rg3 at high dosage on HF was found to be comparable to that of metoprolol. Furthermore, in murine cardiac fibroblasts, the intervention of angiotensin II (AngII) resulted in an upregulation of collagen 1, collagen 3, ɑ-smooth muscle actin, tissue inhibitor of metalloproteinases 1, and the TGF-β1/Smad3 signaling pathway, which could be reversed in case of overexpressing ACY1 and Rg3 administration (Lai et al., 2022). Additionally, Baoyuan decoction, a mixture of several Chinese herbs, consists of *Astragalus membranaceus* (Fisch.) Bunge, *Glycyrrhiza uralensis* Fisch., *Cinnamomum cassia* Presl and *P. ginseng* C. A. Mey. (Wang et al., 2020). A study demonstrated that its active component Rg3 effectively suppressed cardiomyocyte apoptosis via angiotensin type 1 receptor (AT1)-cardiac ankyrin repeat protein (CARP) signaling pathway (Wang et al., 2020). CARP, a downstream protein of AT1 can accelerate apoptosis by activating the P53-mitochondrial apoptotic pathway (Shen et al., 2015). In another study, Rg3 could be an Unc51-like-kinase 1 (ULK1) regulator to facilitate FUN14 domain-containing protein 1 (FUNDC1)-mediated mitophagy, thus restoring mitochondria homeostasis and energy metabolism in HF (Wang et al., 2023). Additionally, trimetazidine was selected as the positive control. The findings indicated that the efficacy of trimetazidine was equivalent to that of a medium dose of Rg3 in rats with HF. Furthermore, a high dose of Rg3 exhibited the most pronounced therapeutic effectiveness (Wang et al., 2023).

### 4.2 Myocardial infarction (MI) and myocardial ischemia-reperfusion injury (MIRI)

MI and MIRI are global health issues characterized by high incidence and mortality rates across different countries. The pathophysiological basis of MI is closely related to mitochondria dysfunction, the depletion of endogenous antioxidants, and lipid peroxidation (Wang and Kang, 2021; Li D. et al., 2023; Cai et al., 2023). Following an acute MI event, clinical intervention typically involves thrombolysis, percutaneous coronary intervention (PCI), and coronary artery bypass grafting (Doenst et al., 2019; Mackman et al., 2020; Sabatine and Braunwald, 2021). These therapeutic strategies are effective in restoring blood flow, alleviating pain, and minimizing myocardial damage. Although these treatments have contributed to a substantial decrease in mortality rates, a subset of patients may experience complications, including hemorrhage and MIRI (Reed et al., 2017; McCarthy et al., 2018; Doenst et al., 2019). It has been observed that an incidence of 10%–25% of recurrent acute MI and a hospital death rate of 6%–14% among MIRI patients following PCI therapy (Wu et al., 2019). In MIRI, several medications, including antiplatelet drugs may extend survival times. However, a significant proportion of these treatments are associated with adverse effects such as bleeding and suboptimal targeting efficiency (Zhang et al., 2022). Additionally, some studies suggest that conventional antiplatelet medications do not markedly improve clinical symptoms (Li et al., 2021). Thus, combining with traditional Chinese medicine may be a promising therapeutic method under virtue of fewer adverse reactions (Tu et al., 2020).

A few studies in animals have proved that Rg3 exerted protective action on MI by promoting mitophagy, and inhibiting apoptosis, myocardial fibrosis as well as inflammation. A study showed that in isoproterenol (ISO)-induced MI mice, Rg3 pretreatment could decrease ROS content in the myocardium, promote autophagy via the AMP-activated protein kinase (AMPK)/acetyl CoA carboxylase (ACC) signal pathway, and inhibit apoptosis (Sun et al., 2020). Another study displayed that in MI rats, Rg3 downregulated the levels of pro-inflammatory cytokines in serum and cardiac tissue such as TNF-α, interleukin-1β (IL-1β), and IL-6 and increased the levels of anti-inflammatory cytokine interleukin-10 (IL-10). The anti-inflammation mechanism of Rg3 is related to the increased expression levels of sirtuin 1 (SIRT1) and decreased expression of p-P65 (Tu et al., 2020). P65 is a crucial part of the nuclear factor κB (NF-κB). SIRT1-deacetylated P65 inhibits NF-κB activation by impeding its nuclear translocation and further restrains the transcription of TNF-α, IL-6, and other inflammatory genes downstream of NF-κB, thereby mitigating the inflammatory response (Chen et al., 2016; Tu et al., 2020). A study showed in MI mice and TGFβ1-stimulated primary cardiac fibroblasts (CFs), Rg3 suppressed CF proliferation along with collagen deposition by inactivation of transforming growth factor beta receptor 1 (TGFBR1)/Smads signaling dose-dependently (Xu et al., 2023). TGFBR1 overexpression partially abolished Rg3’s inhibition on Smad2/Smad3 activation, CFs growth, together with collagen production. In this study, captopril was employed as the positive control. The results indicated that Rg3 improved cardiac function in a dose-dependent manner, with the high dose of Rg3 demonstrating effects comparable to those of captopril (Xu et al., 2023).

In addition, Rg3 exerted a beneficial role in MIRI via inhibiting inflammation, apoptosis, oxidative stress, and attenuating cardiac fibrosis. In MIRI rat models, the use of Rg3 downregulated significantly the levels of inflammatory cytokines in plasma by inhibiting inhibitor of kappa B alpha (IκBα)/NF-κB signal pathway and further improved cardiac function (Zhang et al., 2016; Li et al., 2020). Moreover, Rg3 ameliorated myocardial collagen deposition via the blockage of the TGF-β/Smad signaling pathway and exerted an anti-apoptotic effect via the Akt/endothelial nitric oxide synthase (eNOS) signaling pathway and the B cell lymphoma-2 (Bcl-2)/Bcl2-associated X protein (Bax) pathway (Wang Y. et al., 2015; Li et al., 2020). Hypoxia/reoxygenation (H/R) is a typical approach to generate MIRI injury in cardiocytes (Du et al., 2023). Rg3 administration strongly targeted FoxO3a to decrease the ROS content via the SIRT1/peroxisome proliferators-activated receptor γ coactivator-1α (PGC1-α)/nuclear factor erythroid 2-related factor (Nrf) pathway, and inhibit inflammation through IκBα/NF-κB signal pathway in H9C2 cells induced by H/R (Li et al., 2020).

### 4.3 Cardiotoxicity

Cardiotoxicity is an essential consideration in evaluating whether drugs can be marketed during preclinical trials and is a major reason for treatment withdrawal even after approval (Coelho et al., 2017). Even though some drugs have been used, cardiotoxicity confines their use in clinical practice. Nowadays, a good chunk of drugs, mostly anti-cancer medicine, usually lead to cardiotoxicity. Cancer therapy-induced cardiotoxicity significantly impacts patients mortality, long-term prognosis, and overall quality of life. Anthracyclines play an essential role in cancer therapy, including doxorubicin (DOX), epirubicin, daunorubicin, actinomycin, and valrubicin, have been widely applied to treat multiple types of cancer (Sawicki et al., 2021; Qu et al., 2022). However, their clinical application has been constrained by diverse adverse actions, including cardiotoxicity (Qu et al., 2022). DOX-induced cardiotoxicity involves a complex mechanism, including excessive ROS production, alterations in cell membrane integrity, and apoptosis (Upadhyay et al., 2020; Rawat et al., 2021; Tai et al., 2023). Standard treatments for cardiotoxicity encompass ACEIs, angiotensin receptor blockers (ARBs), beta-blockers, and calcium channel blockers. Research has indicated that ACEI/ARB and beta-blockers may have a positive impact on reducing the long-term health risks associated with anthracycline-induced cardiac dysfunction (Livi et al., 2021). However, previous studies have yielded inconsistent conclusions, possibly related to tumor subtypes and staging. Consequently, there is a need for further investigation into novel medications and complementary therapeutic approaches. A substantial array of natural medicines plays a significant role in mitigating drug-induced cardiotoxicity (Qu et al., 2022).

Traditional Chinese medicine has gained increasing attention for the treatment of various diseases. The protective effects of Rg3 on drug-induced cardiotoxicity have been proved in experiments. Rg3 demonstrated a remarkable ability to inhibit the upregulation of ROS and malondialdehyde, in the meantime, promote superoxide dismutase (SOD), and restore the balance of SOD/glutathione peroxidase in DOX-treated cardiac microvascular endothelial cells and rats through activating the Nrf2/antioxidant response element (ARE) and PI3K/Akt pathway (Wang X. et al., 2015). Furthermore, Rg3 could enhance endothelial function, thus playing a protective impact on cardiac function (Geng et al., 2020).

Another study showed the administration of Rg3 could enhance cardiac function in a dose-dependent manner. Specifically, in cardiotoxicity induced by microcystin, Rg3 acted by downregulating miR-128–3p and upregulating the expression of double minute 4 protein (MDM4), thereby mitigating oxidative stress and reducing cell apoptosis (Zhou and Xia, 2023).

### 4.4 Diabetic cardiomyopathy (DCM)

DCM is a pathophysiological condition induced by diabetes, potentially leading to HF. The diminished performance of the diabetic heart is attributed to multiple factors, including hyperglycemia, elevated fatty acids, and inflammatory cytokines (Dillmann, 2019). Current therapeutic options for DCM primarily comprise sodium-glucose cotransporter 2 inhibitors, glucagon-like peptide-1 receptor agonists, metformin, thiazolidinediones, and dipeptidyl peptidase 4 inhibitors. While clinical trials have confirmed their effectiveness in ameliorating cardiac dysfunction, their ability to fully cure or substantially improve the prognosis of DCM remains limited. Consequently, there is an urgent need to discover novel treatments specifically targeting DCM. A recent research highlights the potential of Rg3 in DCM management (Zhang et al., 2023). Rg3 appears to protect against DCM by modulating glucose and lipid metabolism, achieved through direct binding to peroxisome proliferator-activated receptor γ (PPAR-γ) and stimulating the adiponectin pathway. Additionally, Rg3 reduces proinflammatory cytokines and mitigates mitochondrial dysfunction. In this study, metformin served as a positive control, and varying doses of Rg3 were evaluated in DCM mice models. The findings indicated that high doses of Rg3, similar to metformin, effectively improved body weight, blood glucose, body fat, and serum lipid levels. However, lower doses of Rg3 did not exhibit these benefits. Moreover, high-dose Rg3 significantly decreased serum creatine kinase (CK), creatine kinase-MB (CK-MB), and lactate dehydrogenase (LDH) levels in diabetic mice, indicative of reduced cardiac dysfunction. In contrast, metformin and low-dose Rg3 only partially improved CK and LDH levels. Therefore, high-dose Rg3 demonstrated more comprehensive systemic effects than metformin.

## 5 Pharmacological action of Rg3 in mental disorders

### 5.1 MDD

MDD, commonly referred to as depression, is a long-lasting, recurring, and possibly life-threatening psychiatric illness that impacts up to 20% of the global population (Nabavi et al., 2017). It is usually characterized by diminished self-esteem, cognitive and emotional impairments, reduced energy levels, as well as unexplained pain. Depression has been recognized as one of the primary contributors to the global burden of disease (Akpınar and Karadağ, 2022). The management of depression has become a critical issue for humanity. Primary drugs include serotonin reuptake inhibitors, serotonin-norepinephrine reuptake inhibitors, serotonin modulators, and atypical antidepressants (Chin et al., 2022). Meta-analyses indicate that the efficacy of existing antidepressants is observed in only about half to one-third of patients with depression (Kennedy et al., 2009; Jensen et al., 2023). Moreover, a significant number of patients experience recurrent episodes of depression. Consequently, there is an urgent need to explore and develop new antidepressant medications. Several investigations have demonstrated that Rg3 exhibits a therapeutic effect on depression.

Rg3 could alleviate depression-like behaviors such as anorexia, anhedonia, and decreased social exploration (Kang et al., 2017; You et al., 2017; Zhang et al., 2017). The exact mechanism refers to inhibiting inflammation cytokines such as IL-1β and IL-6, restoring the balance of TRP-KYN metabolism, enhancing cell proliferation, and suppressing apoptosis. In MDD patients and animal models of depression, the TRP-KYN metabolic pathway is over-activated (Parrott et al., 2016). The metabolic processing of neurotoxic KYN initiates depressive-like behavior after peripheral immune activation. However, Rg3 administration could decrease the level of KYN (Kang et al., 2017). Moreover, one extensively supported hypothesis regarding depression is the neurotrophic hypothesis, positing that the pathogenesis of depression is associated with impaired functioning of the BDNF system within the brain (Colucci-D'Amato et al., 2020). BDNF is crucial for neural signal transduction and the facilitation of neuronal plasticity, achieved through its specific binding and subsequent activation of tropomyosin-related kinase B (TrkB) receptors (Moya-Alvarado et al., 2022; Pahlavani, 2023). In the chronic mild stress mouse model, Rg3 markedly enhanced the expression of BDNF and the phosphorylation of cyclic adenosine monophosphate response element binding protein (CREB), thereby mitigating depressive symptoms (You et al., 2017). Meanwhile, fluoxetine was employed as a positive control, revealing that to achieve a comparable antidepressant effect to that of fluoxetine, a higher dosage of Rg3 is necessary (You et al., 2017). Additionally, it was observed that chronic stress exposure markedly decreased body weight. While fluoxetine was effective in mitigating this weight loss, Rg3 exhibited no significant impact on body weight.

### 5.2 Anxiety disorders

Anxiety disorders constitute the most prevalent category of mental illness, typically originating before or during early adulthood. Key characteristics encompass excessive fear and anxiety or avoidance of perceived threats that are persistent and impairing (Penninx et al., 2021). In adults, anxiety prevention has been evaluated in a few trials of selective or indicated prevention (Deady et al., 2017). Effective treatments for anxiety disorders are available, which not only alleviate symptoms of anxiety but also enhance overall quality of life and functioning. Both pharmacotherapy and psychotherapy are regarded as primary approaches to managing anxiety disorders. Studies have demonstrated that routine medications are mildly to moderately effective in treating these disorders, although there is a noted variability in response rates (Penninx et al., 2021). Chinese herbs have shown therapeutic effects on anxiety including Rg3.

Rg3 has shown a beneficial role in GABA_A_ receptor-related anxiety (Lee et al., 2013). GABA_A_ receptors consist of three subunits (α1, β1, γ2). The GABA_A_ receptor is responsible for fast inhibitory synaptic transmission. The principal physiological and pharmacological functions of GABA_A_ receptors encompass the mitigation of anxiety symptoms in patients. Additionally, the γ2 subunit of the GABA_A_ receptor is pivotal in the control and management of human epilepsy (Lee et al., 2013). In a study, Rg3 exhibited enhancing effects on the GABA-induced inward current (I_GABA_) with γ2 subunit in a dependent manner. When the γ2 subunit expression ratio was raised, the degree of the Rg3-induced activation of the GABA_A_ receptor increased (Lee et al., 2013).

### 5.3 Post-traumatic stress disorder (PTSD)

PTSD is a severe psychiatric condition linked to substantial distress and impaired functional capacity (Bryant et al., 2023). Psychological therapies are established as the primary treatment modality for PTSD (Bisson and Olff, 2021). Numerous systematic reviews have consistently affirmed the efficacy of these therapies in addressing PTSD symptoms (Bisson et al., 2013). Nevertheless, the effectiveness of psychological interventions may fluctuate based on individual factors, including the intensity of PTSD. Consequently, pharmacotherapy plays a crucial role in the symptom management of PTSD patients. While common pharmacological treatments have shown encouraging outcomes in diminishing the severity of PTSD symptoms, their overall efficacy remains moderate, and they may precipitate adverse reactions. Therefore, there is an imperative need for the exploration and development of innovative therapeutic strategies.

Rg3 has been reported to have a role in improving fear memory and spatial memory (Sur and Lee, 2022a). The HPA axis and monoamine imbalance in the medial prefrontal cortex and hippocampus contribute to the pathogenesis of PTSD. However, Rg3 administration could be involved in regulating the HPA axis and BDNF-TrkB pathway (Sur and Lee, 2022a). In rats administered with Rg3, a significant decrease in serum corticosterone and adrenocorticotropic hormone levels was observed, alongside a marked increase in BDNF, TrkB, catecholamine, and 5-HT concentrations. The study utilized paroxetine hydrochloride as a positive control. Compared to paroxetine hydrochloride, Rg3 demonstrated superior effects in behavioral tests. Notably, a dosage of 50 mg/kg of Rg3 was effective in elevating 5-HT levels, while significant differences were observed in the response to paroxetine hydrochloride treatment.

### 5.4 ADHD

ADHD is a mental disorder that typically emerges in childhood and is characterized by developmentally inappropriate and impairing inattention, motor hyperactivity, and impulsivity, with difficulties often continuing into adulthood. Medication and behavioral therapies are widely recognized and frequently utilized treatments for ADHD, demonstrating substantial efficacy and notable rates of symptom remission. Furthermore, a recent meta-analysis has revealed a significant preference for herbal medicine as an effective treatment option for ADHD (Dutta et al., 2022). An open-label pilot study demonstrated that the combination of omega-3 and Korean red ginseng may improve ADHD symptoms and cognitive functions including attention, memory, and executive function in children with ADHD (Lee J. et al., 2020). Additionally, YY162, consisting of Rg3 extracted from *P. ginseng* C. A. Mey. and *Ginkgo biloba* L. (Ben-Azu et al., 2022b; Asiwe et al., 2023), exerted protective activity on ADHD through modulating oxidative metabolism and the BDNF/TrkB pathway (Nam et al., 2014). Yet, the mechanism of Rg3 on ADHD requires further research and exploration.

## 6 Rg3 in the coexistence of cardiac diseases and psychiatric disorders

**FIGURE 3:**
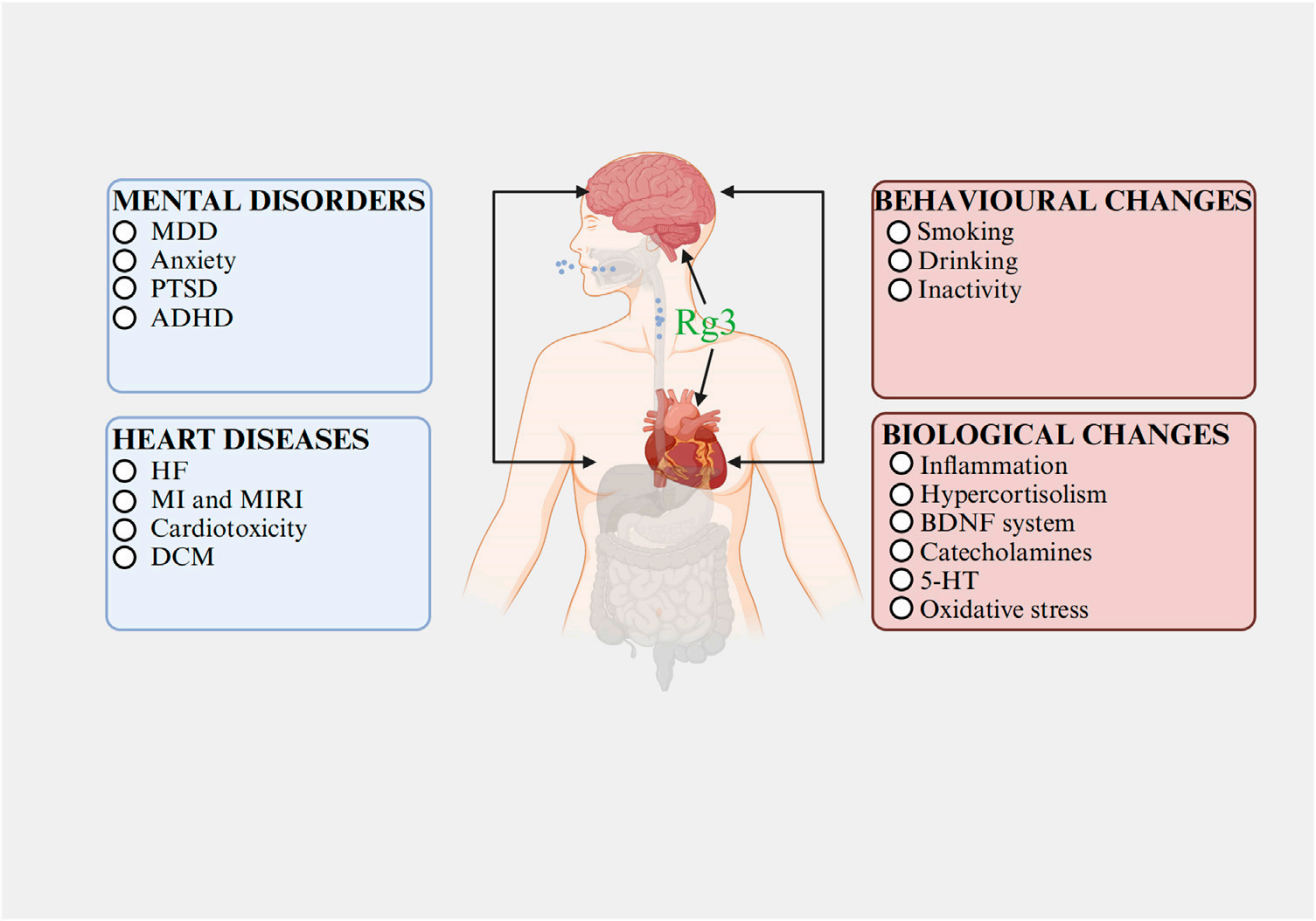
The relationships between heart diseases and mental disorders Abbreviations: MDD, major depressive disorder; PTSD, post-traumatic stress disorder; ADHD, attention deficit hyperactivity disorder; HF, heart failure; MI, myocardial infarction; MIRI, myocardial ischemia-reperfusion injury; DCM, diabetic cardiomyopathy; BDNF, brain-derived neurotrophic factor; 5-HT, serotonin. Image created with BioRender.com, with permission.

Current investigations into the therapeutic effects and mechanisms of Rg3, especially concerning the comorbidity of heart and mental disorders, are still in their early stages. Nowadays, there is substantial basic research supporting Rg3’s efficacy in treating heart or mental disorders separately, based on their relationship, Rg3 may show potential effects in treating their comorbidity (Figure 3).

Rg3 might play a dual preventive role, potentially arresting the progression or aiding the recovery of mental disorders when used in cardiac disease treatments. Its potential mechanisms might involve modulating inflammation and oxidative stress. In cardiac disease management, Rg3 chiefly functions by reducing inflammation and curtailing oxidative stress. Anti-inflammatory action is linked to the regulation of TRP/KYN metabolism, enhancing 5-HT and BDNF levels, and thereby alleviating mental disorders (Kuo et al., 2021; Fellendorf et al., 2022; Gong et al., 2023). MDD is characterized by reduced antioxidant concentrations in plasma (Bhatt et al., 2020). Oxidative stress, characterized by the overproduction of ROS and the depletion of antioxidative defenses, leads to pro-inflammatory signaling and induces cellular apoptosis (Bhatt et al., 2020). Counteracting oxidative stress can therefore diminish inflammation, reduce cellular death, and improve mental health conditions.

Furthermore, in the treatment of mental disorders, Rg3 may also enhance cardiac function. For mental conditions, Rg3 works by suppressing inflammation and HPA axis activity, reducing oxidative stress, boosting BDNF levels, and lowering catecholamine levels. Inflammation and oxidative stress are known to worsen myocardial fibrosis and impair cardiac function. Elevated cortisol levels can directly lead to hypertrophy of cardiomyocytes and cardiac remodeling (Braukyliene et al., 2022). A normal BDNF system is essential for cardiovascular development, while high catecholamine levels can cause vasoconstriction, contributing to cardiac injury, pathological remodeling, heart failure, myocardial ischemia, and necrosis (Du et al., 2021; Agorrody et al., 2022; Tsai et al., 2022). Thus, the mechanism through which Rg3 enhances cardiac function while ameliorating mental disorders may involve modulating inflammation, catecholamine, the BDNF system, the HPA axis, and oxidative stress.

## 7 Adverse reactions of Rg3

Few studies have shown the side effects of Rg3. Existing clinical investigations of Rg3 at various dosages have not revealed any harmful reaction. The sole adverse observation demonstrated increased but reversible kidney weight in dogs that received 60 mg/kg 20(S)-Rg3 (Gao et al., 2020; Nakhjavani et al., 2020). However, given that Rg3 is extracted from ginseng, high doses of ginseng (15 g/d) will lead to ginseng abuse syndrome. Most symptoms encompass headache, dizziness, breast pain, nausea, asthma, and so on (Siegel, 1979; Deng et al., 2023). Although Rg3 offers a broad spectrum of clinical applications in cancer patients, it should not be abused. Taking the recommended dose of Rg3 will not cause serious adverse reactions. Due to the multiple ingredients of ginseng, the adverse effects of Rg3 are not completely equal to ginseng and need to be explored.

## 8 Conclusion and perspectives

**FIGURE 4:**
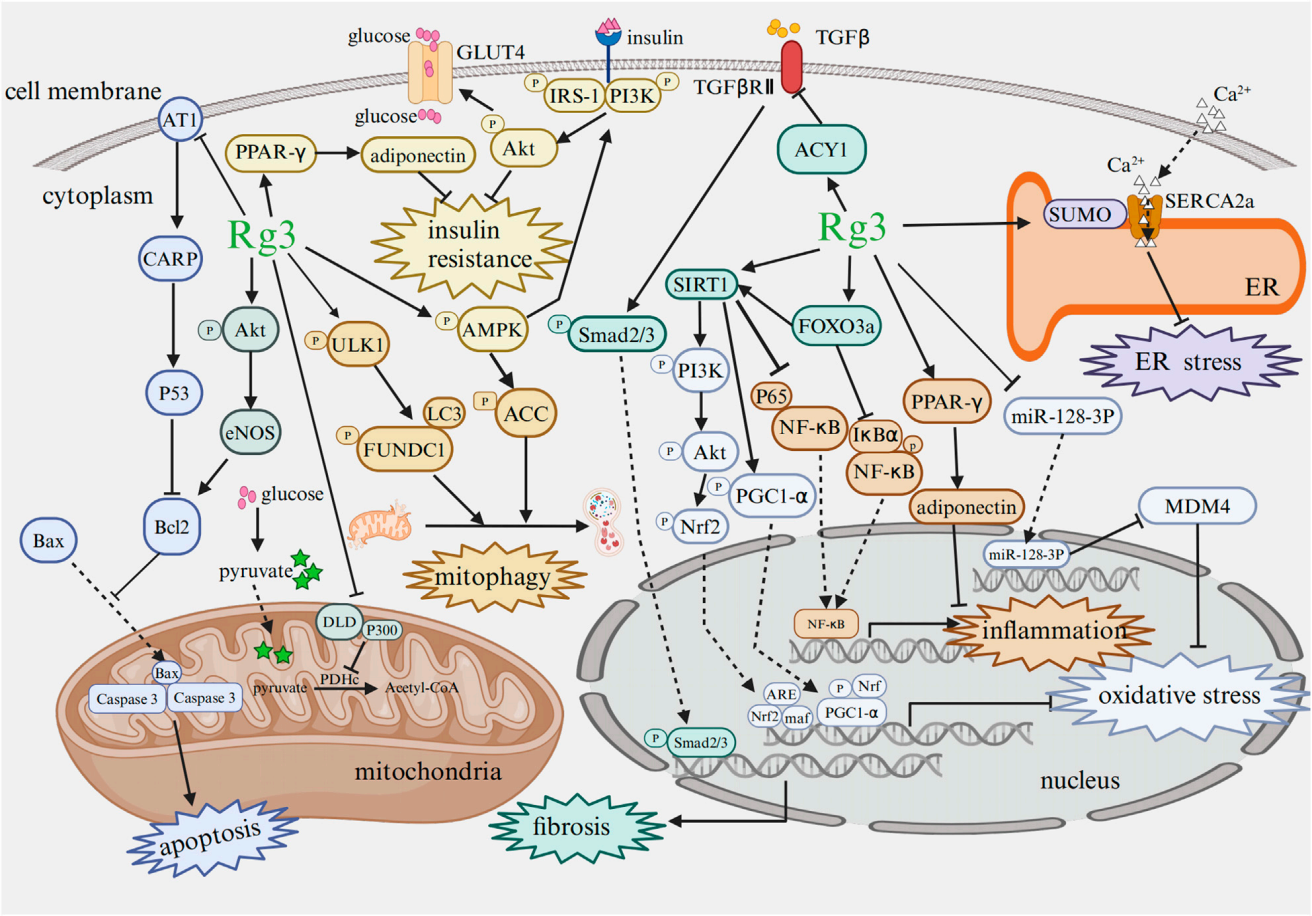
Mechanism of Rg3 on cardiac protection Abbreviations: AT1, angiotensin type 1 receptor; CARP, cardiac ankyrin repeat protein; Bcl2, B cell lymphoma-2; Bax, Bcl2-associated X protein; Akt, protein kinase B; eNOS, endothelial nitric oxide synthase; DLD, dihydrolipoamide dehydrogenase; GLUT4, glucose transporter 4; PDHc, pyruvate dehydrogenase complex; ULK1, Unc51-like-kinase 1; FUNDC1, FUN14 domain-containing protein 1; LC3, microtubule-associated protein 1 light chain 3; PI3K, phosphoinositide 3 kinase; IRS, insulin receptor substrate; AMPK, AMP-activated protein kinase; ACC, acetyl CoA carboxylase; Nrf, nuclear factor erythroid 2-related factor; PGC1-α, peroxisome proliferators-activated receptor γ coactivator-1α; PPAR-γ, peroxisome proliferator-activated receptor γ; NF-κB, nuclear factor κB; IκBα, inhibitor of kappa B alpha; ARE, antioxidant response element; maf, musculoaponeurotic fibrosarcoma oncogene homolog; SIRT1, sirtuin 1; FOXO3a, forkhead box O3a; ER, endoplasmic reticulum; SERCA2a, sarcoplasmic/endoplasmic reticulum Ca^2+^-ATPase; MDM4, double minute 4 protein. Image created with BioRender.com, with permission.

**FIGURE 5:**
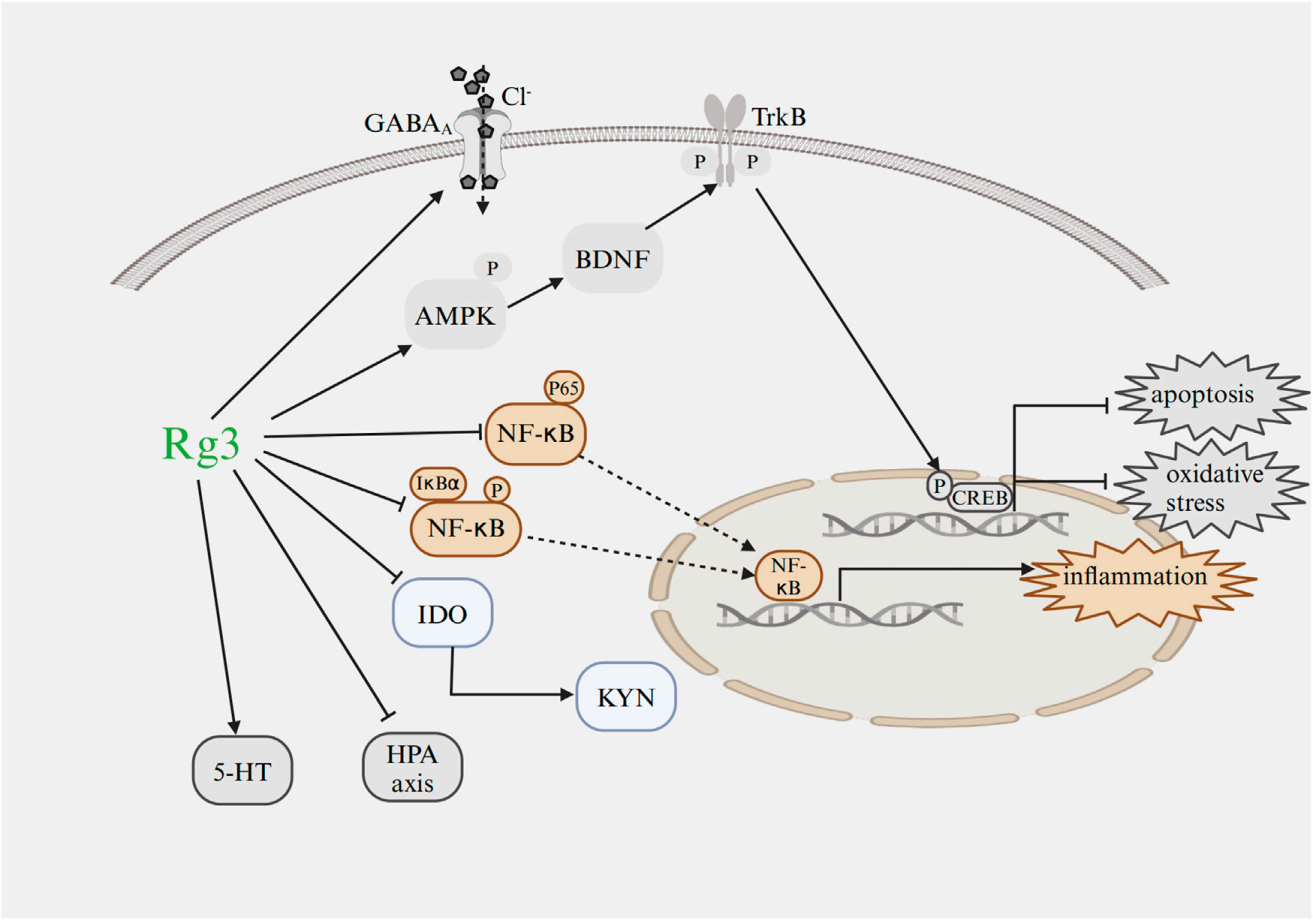
Mechanism of Rg3 on mental disorders Abbreviations: GABA, gamma aminobutyric acid; IDO, indoleamine 2,3-dioxygenase; NF-κB, nuclear factor κB; IκBα, inhibitor of kappa B alpha; KYN, kynurenine; HPA, hypothalamic-pituitary-adrenal; CREB, cyclic adenosine monophosphate response element binding protein; TrkB, tropomyosin-related kinase B; BDNF, brain-derived neurotrophic factor; 5-HT, serotonin; AMPK, AMP-activated protein kinase. Image created with BioRender.com, with permission.

Recent studies have provided increasing evidence of the broad pharmacological effects of Rg3 in treating heart diseases and mental disorders through a variety of signaling pathways and influencing changes at the transcriptional level (Figure 4; Figure 5).

However, the majority of studies primarily concentrate on *in vitro* cell or animal models, with less emphasis on clinical research. The shenyi capsule, comprising Rg3 monomer, is currently employed in the clinical treatment of cancer patients. Clinical studies have demonstrated that shenyi capsules mitigate the toxicity of platinum-based chemotherapy in non-small-cell lung cancer (NSCLC), including a reduction in platelet toxicity (Xu et al., 2020). Additionally, in a double-blind, randomized, crossover study involving 23 participants, oral administration of Rg3-enriched Korean red ginseng at a dose of 400 mg/day for 7 days demonstrated a decrease in aortic stiffness and central blood pressure (Jovanovski et al., 2014). These findings suggest that Rg3 supplementation may help reduce risk factors for heart diseases such as platelet abnormality, vascular stiffness, and hypertension. However, a clinical study was conducted to recruit NSCLC patients with normal cardiac function. The study showed the treatment group with exclusive administration of Rg3 and the control group with placebo treatment exhibited comparable cardiac functions before and after the therapy (Li D.-R. et al., 2023). Furthermore, an open-label pilot study indicated that a combination of omega-3 and Korean red ginseng, which is rich in Rg3, could potentially alleviate symptoms of ADHD (Lee J. et al., 2020).

However, to date, there is no research exploring the exclusive or combined clinical use of Rg3 for treating heart diseases or mental disorders or the comorbidity of both in humans. The limited clinical research on Rg3 may be attributed to several factors. First, existing studies indicate that a higher concentration of Rg3 is necessary to attain similar therapeutic outcomes as routine drugs (You et al., 2017). Then, the utilization of Rg3 in the clinic is restrictive due to low bioavailability and poor intestinal absorption capability with a percentage of approximately 10% (Xiong et al., 2023). Moreover, common methods for preparing Rg3, such as heat treatment, acid-base treatment, and biological transformation (Fu, 2019; Siddiqi et al., 2020), are hindered by the low natural concentration of Rg3 in ginseng plants (Yan et al., 2019). This results in the inability of most methods to achieve large-scale production of Rg3. Finally, there is a necessity to increase awareness and understanding of Rg3’s therapeutic potential in mental disorders and its possible synergistic use in cases of mental disorders co-occurring with heart diseases.

Given these potential reasons, clinical practitioners and researchers are urged to pursue further studies on Rg3. Currently, Rg3 is primarily administered orally. However, novel administration methods, such as catheter-based endocardial or intramyocardial injections, should be explored to enhance its clinical utility. Polyethylene glycol (PEG) is commonly utilized as a hydrophilic block in drug delivery systems for its resistance to protein adsorption and minimal toxicity (Li et al., 2020). Conversely, polypropylene sulfide (PPS) is favored as the hydrophobic block due to its pronounced hydrophobic properties. This PEG-b-PPS amphiphilic block copolymer demonstrates potential as a ROS-responsive nanovesicle, specifically designed for efficient drug delivery (Li et al., 2020). The combination of PEG and PPS offers a promising platform for enhancing the therapeutic effects of Rg3 (Li et al., 2020). Additionally, the development of Rg3-loaded nanoparticles with specific targeting abilities could enable non-invasive, tissue-specific drug delivery (Li et al., 2020). Furthermore, there is also a pressing need for extensive, well-controlled clinical trials to more accurately determine Rg3’s benefits on cardiac and mental health. Regarding Rg3, there exist two variants: 20(R)-Rg3 and 20(S)-Rg3. The distinct impacts of these variants on cardiac and mental health remain to be elucidated. Given that these two epimers have already shown varied anticancer effects, their potential differential influence on heart diseases and mental disorders presents a research opportunity.

In summary, we have reviewed recent findings that illustrate the important role of Rg3 as a novel therapeutic agent in heart diseases and mental disorders. More clinical trials are motivated to be investigated for Rg3 in heart and mental diseases, especially in patients with the coexistence of cardiac diseases and psychiatric disorders.

## Figures and Tables

**Table:** 

**Rg3**	Ginsenoside Rg3
**CAD**	Coronary artery disease
**MDD**	Major depressive disorder
**MI**	Myocardial infarction
**HF**	Heart failure
**MIRI**	Myocardial ischemia-reperfusion injury
**SERCA2a**	Sarcoplasmic/endoplasmic reticulum Ca^2+^-ATPase
**ER**	Endoplasmic reticulum
**ROS**	Reaction oxygen species
**PDHc**	Pyruvate dehydrogenase complex
**DLD**	Dihydrolipoamide dehydrogenase
**ACY1**	Aminoacylase-1
**AngII**	Angiotensin II
**TGF-β1**	Transforming growth factor-β1
**AT1**	Angiotensin type 1 receptor
**CARP**	Cardiac ankyrin repeat protein
**ULK1**	Unc51-like-kinase 1
**FUNDC1**	FUN14 domain-containing protein 1
**PCI**	Percutaneous coronary intervention
**AMPK**	AMP-activated protein kinase
**ACC**	Acetyl CoA carboxylase
**TNF-α**	Tumor necrosis factor-α
**IL-1β**	Interleukin 1 beta
**NF-κB**	Nuclear factor κB
**eNOS**	Endothelial nitric oxide synthase
**Bcl-2**	B cell lymphoma-2
**Bax**	Bcl2-associated X protein
**H/R**	Hypoxia/reoxygenation
**PGC1-α**	Peroxisome proliferators-activated receptor γ coactivator l alpha
**IκBα**	Inhibitor of kappa B alpha
**DOX**	Doxorubicin
**SOD**	Superoxide dismutase
**Nrf**	Nuclear factor erythroid 2-related factor
**ARE**	Antioxidant response element
**PI3K**	Phosphoinositide 3 kinase
**PEG**	Polyethylene glycol
**PPS**	Polypropylene sulfide
**IRS**	Insulin receptor substrate
**TRP**	Tryptophan
**KYN**	Kynurenine
**BDNF**	Brain-derived neurotrophic factor
**CREB**	Cyclic adenosine monophosphate response element binding protein
**I**_**GABA**_	GABA-induced inward current
**PTSD**	Post-traumatic stress disorder
**ADHD**	Attention Deficit Hyperactivity Disorder
**HPA**	Hypothalamic-pituitary-adrenal
**TrkB**	Tropomyosin-related kinase B
**SAM**	Sympathetic-Adrenomedullary System
**MDM4**	Double minute 4 protein
**CK**	Creatine kinase
**CK-MB**	Creatine kinase-MB
**LDH**	Lactate dehydrogenase
**PPAR-γ**	Peroxisome proliferator-activated receptor γ
**TGFBR1**	Transforming Growth Factor Beta Receptor I
**DCM**	Diabetic cardiomyopathy
**SIRT1**	Sirtuin 1
**GABA**	Gamma aminobutyric acid
**ISO**	Isoproterenol
**TIP60**	Tat-interacting protein 60
**Akt**	Protein kinase B
